# [^99m^Tc]Tc-HYNIC-ALUG SPECT/CT in the initial staging of 227 consecutive patients with newly diagnosed prostate cancer: a 5-year monocentric retrospective study

**DOI:** 10.3389/fendo.2024.1326858

**Published:** 2024-02-20

**Authors:** Bo Li, Xintao Ding, Lili Duan, Jingqi Shi, Minmin Tang, Jie Zhang, Zun Zhao, Xinyu Wu, Yongju Gao

**Affiliations:** ^1^ Department of Nuclear Medicine, Henan Key Laboratory of Novel Molecular Probes and Clinical Translation in Nuclear Medicine, Henan Provincial People’s Hospital; Zhengzhou University People’s Hospital, Henan University People’s Hospital, Zhengzhou, China; ^2^ Department of Biomedical Informatics, Columbia University Graduate School of Arts and Sciences, New York, NY, United States

**Keywords:** [99m Tc]Tc-HYNIC-ALUG, SPECT/CT, prostate-specific membrane antigen, primary staging, prostate cancer

## Abstract

**Purpose:**

The purpose of this study was to assess the effectiveness of [^99m^Tc]Tc-HYNIC-ALUG SPECT/CT in the initial staging of patients with newly diagnosed PCa.

**Methods:**

A retrospective analysis was conducted on 227 consecutive patients who underwent [^99m^Tc]Tc-HYNIC-ALUG SPECT/CT imaging for the primary staging of newly diagnosed PCa. The presence and location of PSMA-positive lesions were determined, and the maximum standardized uptake values (SUVmax) of the primary prostate tumor were also measured. The metastatic findings and SUVmax were stratified according to International Society of Urological Pathology (ISUP) grade, prostate-specific antigen (PSA) levels, and D’Amico classification. Furthermore, the [^99m^Tc]Tc-HYNIC-ALUG SPECT/CT findings were compared to the histopathological findings in patients who had undergone radical prostatectomy with pelvic lymph node dissection (PLND).

**Results:**

Of the 227 patients, 92.1% (209/227) had positive [^99m^Tc]Tc-HYNIC-ALUG SPECT/CT findings. Advanced disease was detected in 38.8% (88/227) of the patients and was positively correlated with increasing ISUP grade and PSA levels. Lymph node metastases (both pelvic and extrapelvic), bone metastases, and visceral metastases were detected in 30.0% (68/227), 25.6% (58/227), and 3.1% (7/227) of the patients, respectively. For the 129 patients who underwent radical prostatectomy with PLND, the sensitivity of [^99m^Tc]Tc-HYNIC-ALUG SPECT/CT in the evaluation of PCa was 90.7% (117/129). The sensitivity, specificity, accuracy, and positive and negative predictive values for detecting pelvic lymph node metastases on [^99m^Tc]Tc-HYNIC-ALUG SPECT/CT were 23.5% (12/51), 93.6% (73/78), 65.9% (85/129), 70.6% (12/17), and 65.2% (73/112), respectively. Among the 209 patients with PSMA-avid primary prostate disease, the SUVmax of the primary prostate tumor was significantly associated with ISUP grade (p<0.0001), PSA levels (p<0.0001), D’Amico classification (p<0.0001), and advanced disease (p<0.0001). Receiver operating characteristic (ROC) analysis revealed that a PSA level >19.8 ng/ml and SUVmax of the primary prostate tumor >7.4 had a sensitivity of 71.6% and 71.6% and specificity of 76.9% and 82.6%, respectively, for detecting metastatic disease.

**Conclusions:**

[^99m^Tc]Tc-HYNIC-ALUG SPECT/CT emerges as a valuable imaging tool for the initial staging of newly diagnosed PCa. The presence of advanced disease and the SUVmax of the primary prostate tumor were positively correlated with ISUP grade and PSA levels.

## Introduction

Prostate cancer (PCa) is the second most commonly diagnosed malignancy and the fifth leading cause of cancer-related mortality among men globally (1, 2). Therefore, accurate primary staging in PCa is crucial for devising treatment strategies and predicting patient outcomes (3). Conventional imaging modalities such as computed tomography (CT), magnetic resonance imaging (MRI), and bone scintigraphy have traditionally been employed for the primary staging of PCa. CT and MRI provide morphological information, mainly based on morphological changes (such as irregular shape or enlarged lymph node), but they show limited sensitivity for the early detection of metastatic disease. Bone scintigraphy, on the other hand, is highly sensitive but has low specificity for detecting bone metastases in PCa (4).

Prostate-specific membrane antigen (PSMA), also known as glutamate carboxypeptidase II, is a transmembrane glycoprotein prominently overexpressed in PCa cells. Therefore, PSMA has recently gained increasing interest as a promising target for PCa diagnosis and treatment with radionuclides (5). PSMA targeting was achieved using small molecule urea-based PSMA inhibitor. The most widely used PET tracers for primary staging of newly diagnosed PCa patients are PSMA inhibitors coupled with [^68^Ga]Ga, such as [^68^Ga]Ga-PSMA-11 and [^68^Ga]Ga-PSMA-617, which have shown superior diagnostic performance compared to conventional imaging techniques. A systematic review published by Satapathy et al. (6) demonstrated the excellent sensitivity of [^68^Ga]Ga-PSMA-11 and [^68^Ga]Ga-PSMA-617 PET/CT for initial detection in patients with suspected PCa. Ergül et al. (7) demonstrated that [^68^Ga]Ga-PSMA-11 PET/CT is a highly effective imaging modality for the initial evaluation of newly diagnosed PCa, leading to significant changes in its staging compared to conventional imaging methods. In addition to the [^68^Ga]Ga-labeled PSMA radiotracers, Chikatamarla et al. (8) demonstrated comparable superior diagnostic accuracy for primary staging of PCa using [^18^F]F-labeled PSMA inhibitor ([^18^F]PSMA-1007) in their largest study.

Although PSMA-targeted PET tracers are promising, [^99m^Tc]Tc-labeled small-molecule PSMA inhibitors have emerged as an attractive alternative due to the widespread availability of ^99^Mo/^99m^Tc generators and SPECT/CT devices, especially in medical institutions where PET/CT is not available. Several [^99m^Tc]Tc-labeled PSMA inhibitors, including [^99m^Tc]Tc-MIP-1404, [^99m^Tc]Tc-PSMA-T4, [^99m^Tc]Tc-PSMA-I&S, and [^99m^Tc]Tc-EDDA/HYNIC-iPSMA, have been developed for PCa detection (9–12). Previously, Ferro-Flores et al. (13) demonstrated that [^99m^Tc]Tc-EDDA/HYNIC-iPSMA is as effective as [^68^Ga]Ga-PSMA-617 in diagnosing PCa tumors and their metastases. A prospective study conducted by García-Pérez et al. (11) reported comparable results between [^68^Ga]Ga-PSMA-11 PET/CT and [^99m^Tc]Tc-EDDA/HYNIC-iPSMA SPECT/CT in patients with progressive metastatic PCa. Recently, a novel HYNIC-modified small molecule PSMA inhibitor, 6-hydrazinonicotinate-Aminocaproic acid-Lysine-Urea-Glutamate (HYNIC-ALUG), has been developed (14). [^99m^Tc]Tc-HYNIC-ALUG exhibits specific accumulation in PSMA-positive tumors and low dosimetry (14, 15). Moreover, the labeling method is simpler, faster, and does not require further purification, making it suitable for routine clinical applications. Our previous study demonstrated the effectiveness of [^99m^Tc]Tc-HYNIC-ALUG SPECT/CT in detecting biochemically recurrent PCa after radical prostatectomy (16).

The majority of studies assessing [^99m^Tc]Tc-HYNIC-ALUG imaging have been reported in PCa patients with biochemical recurrence. However, studies on the initial evaluation and staging of newly diagnosed PCa using [^99m^Tc]Tc-HYNIC-ALUG are scarce. Therefore, this study aimed to investigate the role of [^99m^Tc]Tc-HYNIC-ALUG SPECT/CT in the primary staging of PCa and its correlation with traditional risk stratification factors, including histological biopsy International Society of Urological Pathology (ISUP) grade, PSA level, and maximum standardized uptake value (SUVmax) of the primary prostate tumor. Additionally, we assessed the correlation between [^99m^Tc]Tc-HYNIC-ALUG SPECT/CT findings and histopathological results in patients who underwent radical prostatectomy (RP) with pelvic lymph node dissection (PLND).

## Patients and methods

### Study design and population

A retrospective analysis was performed between November 2018 and June 2023 on 227 consecutive patients who underwent [^99m^Tc]Tc-HYNIC-ALUG SPECT/CT scan for primary staging of newly diagnosed PCa at the Department of Nuclear Medicine, Henan Provincial People’s Hospital & Zhengzhou University People’s Hospital. All patients enrolled were in the initial staging phase and naïve to therapy, including androgen deprivation therapy. Demographic information, PSA levels, and ISUP grade were collected for each patient. Based on the PSA level and ISUP grade, risk groups were stratified according to the D’Amico classification. Low risk was defined as PSA <10 ng/mL and ISUP grade 1, intermediate risk as PSA 10-20 ng/mL or ISUP grades 2 and 3, and high risk as PSA >20 ng/mL or ISUP grades 4 and 5. Additionally, 129 patients underwent RP with PLND. The proportion of patients with a change in ISUP grade after RP was recorded. Furthermore, the number of resected lymph nodes and the number of pathologically confirmed malignant lymph nodes were also recorded.

The Institutional Review Board of Henan Provincial People’s Hospital & Zhengzhou University People’s Hospital approved this study. Informed consent was waived because of the retrospective nature of the study, and the analysis used anonymous clinical data.

### [^99m^Tc]Tc-HYNIC-ALUG SPECT/CT

[^99m^Tc]Tc-HYNIC-ALUG was synthesized using previously described procedures (14, 17). The radiochemical purity of the [^99m^Tc]Tc-HYNIC-ALUG produced was greater than 95%, as determined by high-performance liquid chromatography (HPLC). All [^99m^Tc]Tc-HYNIC-ALUG SPECT/CT scans were performed 3-4 hours after following the intravenous injection of 10 MBq of [^99m^Tc]Tc-HYNIC-ALUG per kilogram of body weight. Planar whole-body scintigraphy and SPECT/CT imaging were acquired with a dual-headed SPECT/CT gamma camera (Symbia T16; Siemens Healthcare, Erlangen, Germany) using a low-energy high-resolution collimator. Anterior and posterior planar whole-body scans were performed at a speed of 12 cm/min with a matrix size of 256 × 1024 and a zoom factor of 1.

The quantitative SPECT/CT images, spanning from the neck to the proximal thighs, were obtained immediately following the planar scan. Additionally, in instances where abnormal PSMA uptake was observed beyond the routine scanning range, supplementary SPECT/CT scans of the relevant regions were conducted. The SPECT imaging parameters were as follows: matrix size 256 × 256; angular resolution of 6 degrees in 30 steps; and acquisition time 30 seconds per step. SPECT data were quantitatively reconstructed with Siemens xSPECT-Quant, which includes scatter compensation and attenuation corrections (16). The scan parameters for low-dose CT were 130 kV and 25 reference mAs, and images were reconstructed with a 5-mm slice thickness using a B31s medium smooth reconstruction kernel (Siemens Healthcare).

### Image analysis

All [^99m^Tc]Tc-HYNIC-ALUG SPECT/CT scans were independently interpreted by two experienced nuclear medicine physicians, and a final diagnosis was determined by consensus. The nuclear medicine physicians were blinded to the clinical and laboratory results. Images were analyzed at a workstation with the commercial fusion software “syngo” (Siemens, Medical Healthcare, Erlangen, Germany), which provided multiplanar reformatted images and displayed SPECT, CT and fusion images. A PSMA-positive lesion was defined as a focal [^99m^Tc]Tc-HYNIC-ALUG avidity higher than the surrounding background and not associated with physiologic uptake. The locations of PSMA-avid lesions were classified into intraprostatic disease, pelvic lymph node metastases (N1), extrapelvic lymph node metastases (M1a), bone metastases (M1b), and visceral metastases (M1c). Among them, extraprostatic PSMA-positive lesions were considered advanced disease (N1 and M1 disease). For quantitative analysis of PSMA-positive primary prostate tumors, SUVmax was obtained by volume of interest (VOI) with isocontours set at 40% of maximum uptake within the prostate.

### Statistical analysis

Patient and clinical characteristics were summarized using count and percentage for categorical variables, mean and standard deviation (SD) for normally distributed continuous variables, median and interquartile range (IQR) for non-normally distributed continuous variables. The percentage of metastatic findings and the SUVmax of the primary prostate tumor were plotted against the ISUP grade, PSA level, and D’Amico classification. To assess differences between groups, we used Chi-square tests for categorical variables, Student t tests for normally distributed continuous variables, and Wilcoxon rank-sum tests for non-normally distributed continuous variables. P values less than 0.05 considered statistically significant. Sensitivity, specificity, accuracy, and positive and negative predictive values of [^99m^Tc]Tc-HYNIC-ALUG SPECT/CT were calculated using histopathology results as the reference standard. Receiver operating characteristic (ROC) curve analysis and the area under the ROC curve (AUC) with 95% confidence intervals (CIs) were calculated to evaluate the predictive ability of PSA levels and SUVmax of the primary prostate tumor in predicting advanced disease on [^99m^Tc]Tc-HYNIC-ALUG SPECT/CT. The violin plot was generated using the R package ggplot2. All statistical analyses were performed using R software (version 3.5.3).

## Results

### Patient characteristics

The demographic information, PSA values, ISUP grade, and D’Amico risk group are illustrated in **Table 1**. A total of 227 consecutive patients who underwent [^99m^Tc]Tc-HYNIC-ALUG SPECT/CT imaging for primary staging of newly diagnosed PCa were retrospectively analyzed. The median age was 71.1 years (range 46-90) at the time of [^99m^Tc]Tc-HYNIC-ALUG SPECT/CT imaging. The median PSA level before [^99m^Tc]Tc-HYNIC-ALUG SPECT/CT was 26.1 ± 24.3 ng/ml (range: 0.5-188.7 ng/ml). Based on the PSA levels, there were 61 (26.9%) patients with PSA < 10 ng/ml, 76 (33.5%) patients with PSA 10-20 ng/ml, and 90 (39.6%) patients with PSA > 20 ng/ml. The pathology of biopsies revealed ISUP 1 in 43 (18.9%) patients, ISUP 2 in 30 (13.2%) patients, ISUP 3 in 55 (24.2%) patients, ISUP 4 in 41 (18.1%) patients, and ISUP 5 in 58 (25.6%) patients. According to the D’Amico risk classification system, 18 (7.9%), 88 (38.8%) and 121 (53.3%) patients were classified in the low-, intermediate-, and high-risk groups, respectively.

**Table 1:** Clinical characteristics of 227 patients.

Characteristic		Value
Age at SPECT/CT (years)		71.1 (range: 46–90)
PSA level (ng/ml)	<10	61 (26.9%)
	10-20	76 (33.5%)
	>20	90 (39.6%)
	Mean ± SD	26.1 ± 24.3 (range: 0.5-188.7)
ISUP grade	1	43 (18.9%)
	2	30 (13.2%)
	3	55 (24.2%)
	4	41 (18.1%)
	5	58 (25.6%)
D’Amico risk group	Low	18 (7.9%)
	Intermediate	76 (38.8%)
	High	133 (53.3%)
Localization of PSMA positive lesions*	Prostate region	209 (92.1%)
	Lymph node metastases	68 (30.0%)
	Bone metastases	58 (25.6%)
	Visceral metastases	7 (3.1%)
Advanced disease	No	139 (61.2%)
	Yes	88 (38.8%)

*More than 1 region could be involved per patient.

### Location of PSMA-positive lesions

**Figure 1:**
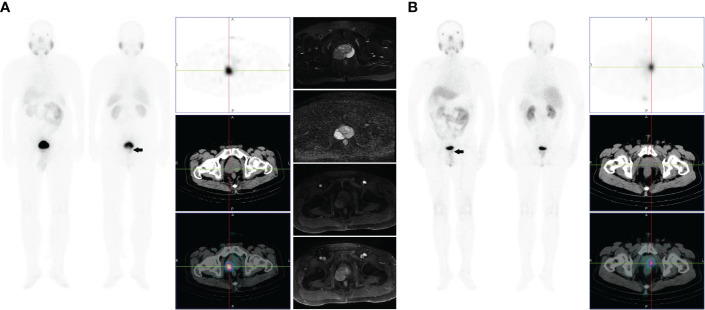
**(A)** Images of a 67-year-old male with high-risk prostate cancer (PSA, 11.3 ng/ml; ISUP grade 5). Whole-body planar scintigraphy (indicated by the black arrow) and transaxial SPECT/CT fusion images showed a solitary PSMA avid focus within the right mid posterolateral prostate gland (SUVmax 5.3). Plain and enhanced magnetic resonance imaging scans showed abnormal signals in the right peripheral zone, which are indicative of prostate cancer. **(B)** Images of a 61-year-old male with intermediate-risk prostate cancer (PSA, 8.4 ng/ml; ISUP grade 3). Whole-body planar scintigraphy (indicated by the black arrow) and transaxial SPECT/CT fusion images showed a solitary PSMA avid focus within the left mid-posterolateral prostate gland (SUVmax 4.5).

**Figure 2:**
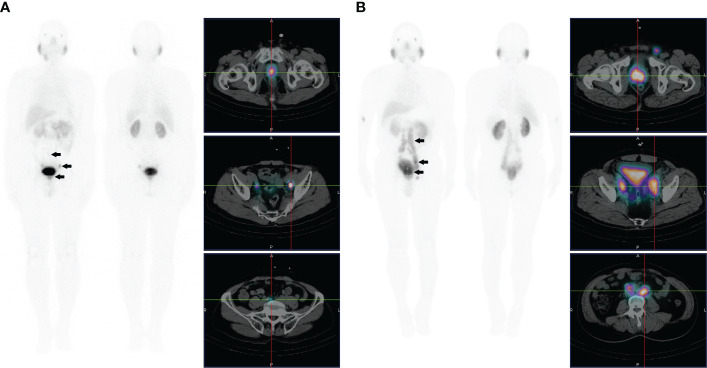
**(A)** Images of a 78-year-old male with high-risk prostate cancer (PSA, 23.9 ng/ml; ISUP grade 3). Whole-body planar scintigraphy (indicated by the black arrow) and transaxial SPECT/CT fusion images showed abnormally increased PSMA uptake in prostate cancer (SUVmax 5.1) and multiple PSMA-avid lesions in lymph nodes adjacent to the bilateral iliac vessels of the pelvic cavity. **(B)** Images of a 66-year-old male with high-risk prostate cancer (PSA, 63.2 ng/ml; ISUP grade 5). Whole-body planar scintigraphy (indicated by the black arrow) and transaxial SPECT/CT fusion images showed abnormally increased PSMA uptake in prostate cancer (SUVmax 13.6) and multiple PSMA-avid lesions in pelvic and extrapelvic lymph nodes.

**Figure 3:**
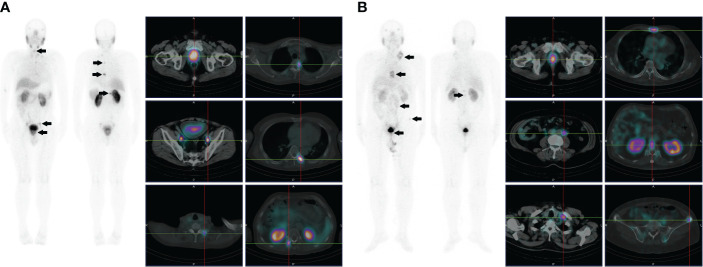
**(A)** Images of a 72-year-old male with high-risk prostate cancer (PSA, 188.7 ng/ml; ISUP grade 5). Whole-body planar scintigraphy (indicated by the black arrow) and transaxial SPECT/CT fusion images showed abnormally increased PSMA uptake in prostate cancer (SUVmax 22.5) and multifocal PSMA avid distant metastatic disease (including pelvic lymph node and bone metastases). **(B)** Images of a 68-year-old male with high-risk prostate cancer (PSA, 90.3 ng/ml; ISUP grade 5). Whole-body planar scintigraphy (indicated by the black arrow) and transaxial SPECT/CT fusion images revealed abnormally increased PSMA uptake in prostate cancer (SUVmax 13.1) and multifocal PSMA-avid distant metastatic disease (including pelvic and extrapelvic lymph nodes and bone metastases).

Among the 227 patients, [^99m^Tc]Tc-HYNIC-ALUG SPECT/CT detected at least one positive lesion in 209 (92.1%) patients but was negative in 18 (7.9%) patients. Prostate primary PSMA-avid diseases were detected in 92.1% (209/227) of patients (**Figure 1**), lymph node metastases were detected in 30.0% (68/227) (**Figures 2**, **3**), bone metastases were detected in 25.6% (58/227) (**Figure 3**), and visceral metastases were detected in 3.1% (7/227) of the patients. Advanced disease was detected in 38.8% (88/277) of patients (**Figures 2**, **3**). Among them, 53.4% (47/88) had single organ metastases, while 46.6% (41/88) had multiple organ metastases. **Table 1** lists the different regions where PSMA-positive lesions occurred.

### Lymph node metastases

**Figure 4:**
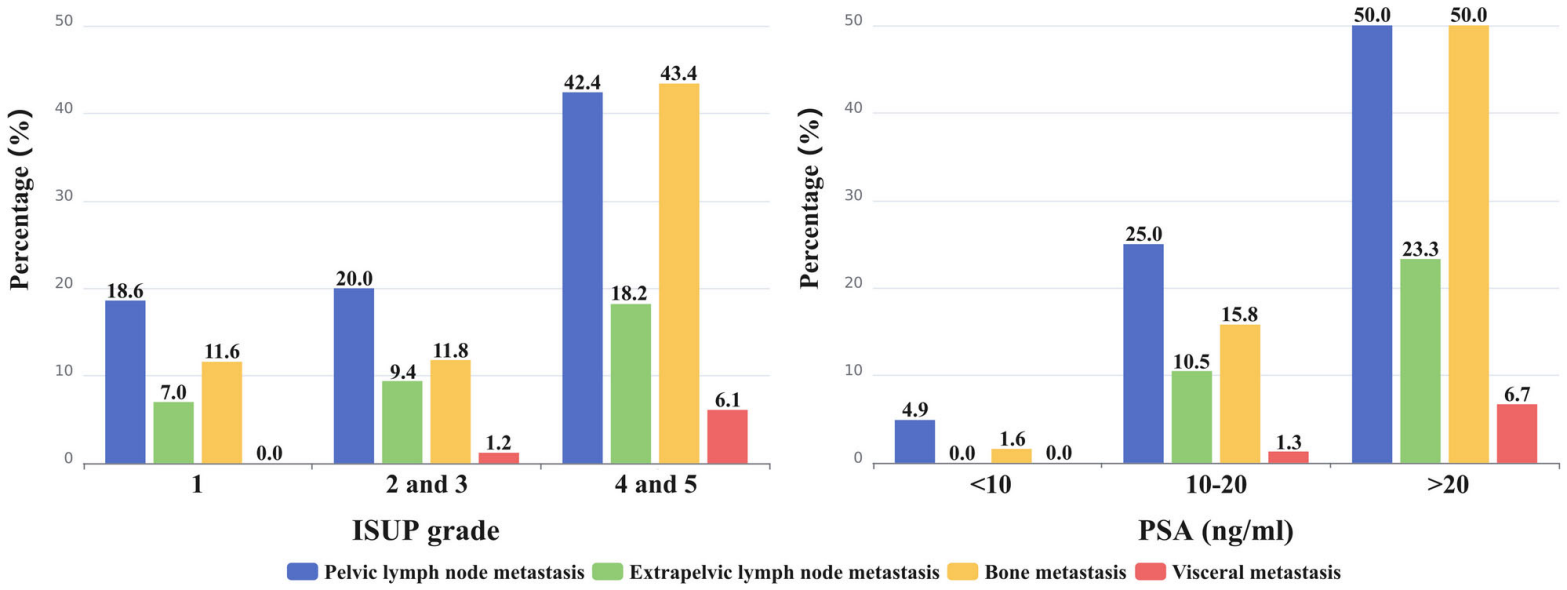
The percentage of metastasis to different sites (pelvic and extrapelvic lymph node metastases, bone metastasis, and visceral metastases) was calculated and represented on the bar graph.

As depicted in **Figure 4**, ISUP 1 had 18.6% (8/43) of pelvic nodal metastases, ISUP 2 and 3 had 20.0% (17/85), and ISUP 4 and 5 had 42.4% (42/99). The percentage of patients with extrapelvic lymph node metastases were 7.0% (3/43) for ISUP 1, 9.4% (8/85) for ISUP 2 and 3, and 18.2% (18/99) for ISUP 4 and 5. As the ISUP grade increased, there was a significant increase in the presence of pelvic lymph node metastases (p=0.0009). Although the prevalence of extrapelvic nodal metastases also increased with higher ISUP grade, the difference was not statistically significant (p=0.0927).

Similarly, increasing PSA levels were associated with an increased risk of pelvic (p<0.0001) and extrapelvic (p=0.0001) nodal metastases (**Figure 4**). Pelvic lymph node metastases were detected in 4.9% (3/61) of patients with a PSA level <10 ng/mL, in 25.0% (19/76) of patients with a PSA level of 10-20 ng/mL, and in 50.0% (45/90) of patients with a PSA level >20 ng/mL. Notably, no extrapelvic lymph node metastases were observed in patients with a PSA level <10 ng/mL. However, in patients with a PSA level of 10-20 ng/mL, the incidence was 10.5% (8/76), and in those with a PSA level >20 ng/mL, it increased to 23.3% (21/90).

Based on the D’Amico classification system, lymph node metastasis (pelvic and extrapelvic) was not observed in any patient in the low-risk group but was detected in 9 (10.2%) patients in the intermediate-risk group and 59 (48.8%) patients in the high-risk group. Compared with the intermediate-risk group, high-risk patients had a significantly higher proportion of lymph node metastases (P<0.0001).

### Bone and visceral metastasis

As shown in **Figure 4**, bone metastasis was detected in 11.6% (5/43), 11.8% (10/85), and 43.4% (43/99) of patients in ISUP 1, ISUP 2 and 3, and ISUP 4 and 5, respectively. PSMA avid bone metastasis was detected in 1 (1.6%) patient with PSA levels of <10 ng/mL, in 12 (15.8%) patients with PSA levels of 10-20 ng/mL, and in 45 (50.0%) patients with PSA levels of >20 ng/mL. Bone metastasis was more likely to occur in PCa patients with a higher ISUP grade (ISUP 4 and 5) (P<0.0001) and PSA level (PSA>20 ng/mL) (P<0.0001). Consequently, the proportion of bone metastases occurring in the high-risk group (44.6%; 54/121) was significantly higher than that in the intermediate-risk group (4.5%; 4/88) (P<0.0001).

PSMA-avid visceral metastases were absent in ISUP 1; 1.2% (1/85) of visceral metastases were detected in ISUP 2 and 3, whereas 6.1% (6/99) were detected in ISUP 4 and 5. In patients with PSA levels <10 ng/mL, there were no visceral metastases. In patients with PSA levels of 10-20 ng/mL, there was a 1.3% (1/76) occurrence, while in patients with PSA levels >20 ng/mL, the percentage rose to 6.7% (6/90). As the ISUP grade (p=0.0952) and PSA level (p=0.0598) increased, the presence of visceral metastases increased. All 7 patients with visceral metastases were present in the high-risk group.

### Diagnostic accuracy of PSMA SPECT/CT

A total of 129 patients underwent subsequent RP and PLND. All RP specimens were pathologically confirmed to be PCa. Among them, [^99m^Tc]Tc-HYNIC-ALUG SPECT/CT imaging was positive in 117 patients but negative in 12 patients. Accordingly, the sensitivity of [^99m^Tc]Tc-HYNIC-ALUG SPECT/CT in the evaluation of PCa was 90.7% (117/129). Since all the patients had histologically confirmed PCa, specificity could not be calculated. Interestingly, the biopsy ISUP grade of 32 patients was downgraded, and 17 patients were upgraded after RP pathology. The pelvic lymph nodes resected from these patients were also histologically evaluated. A total of 41 PSMA-positive lymph nodes were found in 17 patients. Twelve patients were pathologically confirmed to have lymph node metastases, whereas 5 patients were confirmed to be false positives. Of the 112 [^99m^Tc]Tc-HYNIC-ALUG SPECT/CT-negative patients, 73 patients were considered true negatives, and 39 patients were false negatives. Hence, the sensitivity, specificity, diagnostic accuracy, positive predictive value, and negative predictive value of [^99m^Tc]Tc-HYNIC-ALUG SPECT/CT imaging in detecting pelvic lymph node metastasis were 23.5% (12/51), 93.6% (73/78), 65.9% (85/129), 70.6% (12/17), and 65.2% (73/112), respectively (**Table 2**).

**Table 2:** A concordance between PSMA-positive or PSMA-negative lymph nodes and pathological verification was assessed in 129 patients undergoing radical prostatectomy with pelvic lymph node dissection.

PSMA SPECT/CT	Histology		Index
	Positive,n (%)	Negative,n (%)	
Positive,n (%)	12 (9.3)	5 (3.9)	PPV=70.6%
Negative,n (%)	39 (30.2)	73 (56.6)	NPV=65.2%
Index	Se=23.5%	Sp=93.6%	Ac=65.9%

Se, sensitivity; Sp, specificity; PPV, positive predictive value; NPV, negative predictive value; Ac, Accuracy.

For the 39 patients with no detected lymph node metastasis on [^99m^Tc]Tc-HYNIC-ALUG SPECT/CT imaging, we observed a significantly lower proportion of high-risk patients in this subgroup compared to PSMA-detected patients (46.2% vs. 66.7%; p=0.0030). Both groups had no low-risk patients. There were no significant statistical differences in PSA levels (p=0.2210) and GS scores (p=0.4902) between the two groups. Additionally, when comparing the pathological sizes of lymph nodes between the two groups, we found that the average diameter of lymph nodes in the detected group was larger than in the undetected group (17.7 ± 7.8 vs. 10.1 ± 3.0 mm; p=0.0045).

### Measurements of SUVmax in primary prostate tumors

**Figure 5:**
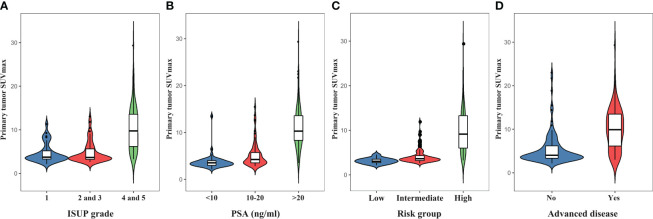
The violin plots indicate increasing primary prostate tumor SUVmax with higher ISUP grades **(A)**, PSA levels **(B)**, D’Amico risk groups **(C)**, and the presence of advanced disease **(D)**.

Due to negligible PSMA uptake in the primary prostate tumors, 18 patients were excluded from the SUVmax analysis. The SUVmax of the primary tumor ranged from 2.16 to 29.39, with a mean of 7.40 ± 4.78. The SUVmax in relation to ISUP grades and PSA levels is depicted in **Figure 5**. The median SUVmax of the primary tumor was 4.62 ± 2.19 in ISUP 1, 4.81 ± 2.44 in ISUP 2 and 3, and 10.46 ± 5.07 in ISUP 4 and 5. For PSA <10, 10-20, and >20 ng/mL, the median SUVmax was 3.74 ± 1.60, 5.29 ± 2.68, and 11.18 ± 4.68, respectively. According to the results, both the ISUP grade (p<0.0001) and PSA level (p<0.0001) were significantly associated with the SUVmax. Moreover, the high-risk group (9.88 ± 4.89) had a higher median SUVmax than the intermediate-risk group (4.28 ± 1.77) and low-risk group (3.15 ± 0.65) (p<0.0001). Accordingly, the SUVmax of primary tumors was significantly higher in patients with advanced disease than in patients with localized disease (10.14 ± 4.89 vs. 5.40 ± 3.60; p<0.0001).

### Prediction of metastasis with PSA level and SUVmax

**Figure 6:**
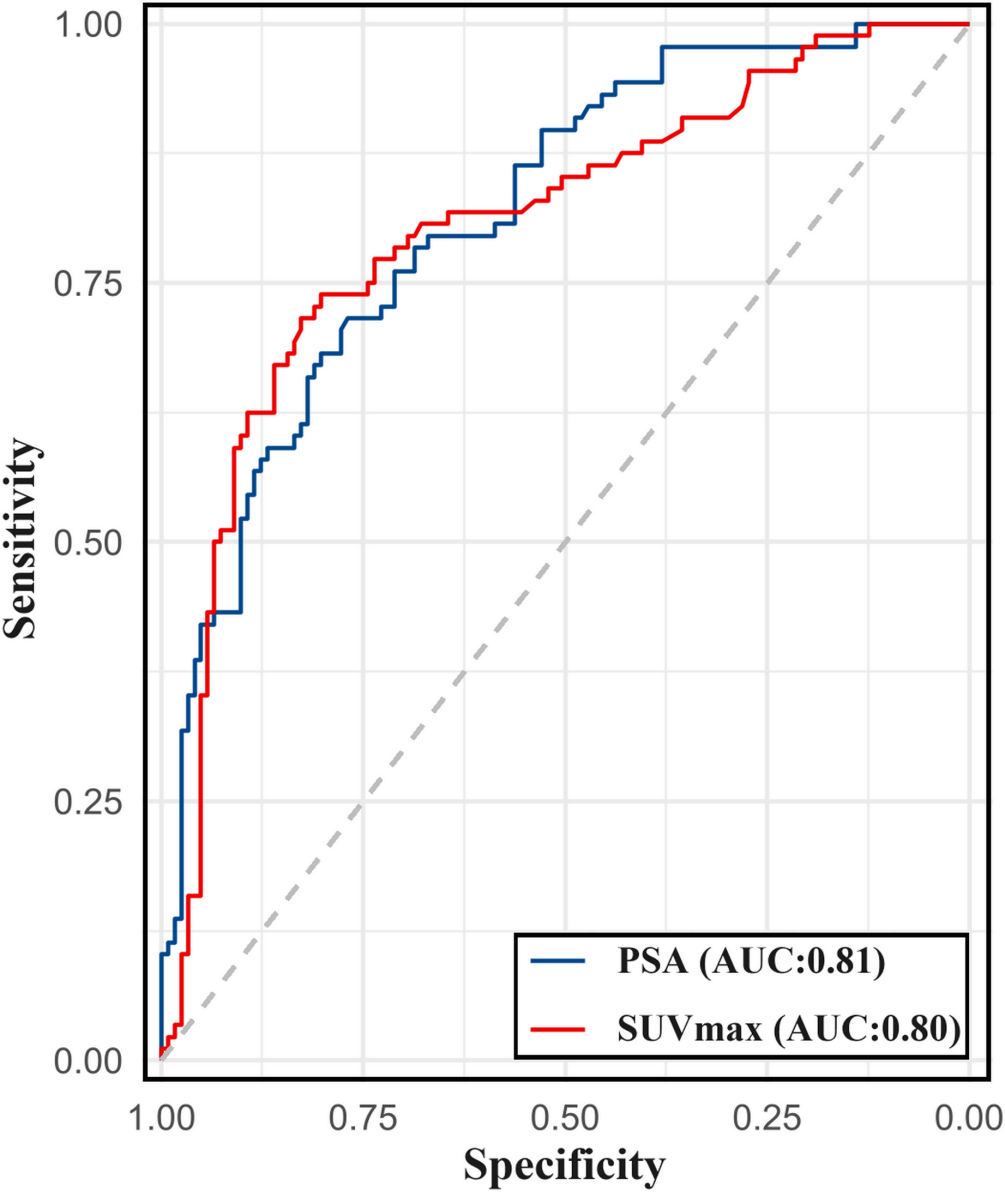
The ROC curve for PSA levels and SUVmax of primary prostate tumor in predicting metastasis on [^99m^Tc]Tc-HYNIC-ALUG SPECT/CT.

A ROC curve analysis was conducted to predict the presence of metastatic lesions on [^99m^Tc]Tc-HYNIC-ALUG SPECT/CT using the PSA level and SUVmax of the primary prostate tumor. As shown in **Figure 6**, the AUC (95% CI) for PSA and SUVmax were 0.81 (0.76–0.87) and 0.80 (0.74–0.87), respectively.

The ROC analysis for PSA level determined an optimal cutoff value of 19.8 for discriminating between patients with and without metastatic disease, with a sensitivity of 71.6% and specificity of 76.9%. For SUVmax, the optimal cutoff value was determined to be 7.4 for discriminating between patients with and without metastatic disease, with a sensitivity of 71.6% and specificity of 82.6%. For ISUP grade >3, the sensitivity for detecting metastatic disease using [^99m^Tc]Tc-HYNIC-ALUG SPECT/CT was 64.8%, with a specificity of 66.9%.

As depicted in **Table 3**, the sensitivity for detecting metastatic disease was 60.2%, with a specificity of 84.3% when combining SUVmax >7.4 with PSA >19.8 ng/ml. The sensitivity and specificity for the combination of SUVmax >7.4 with ISUP >3 were 58.0% and 87.6%, respectively.

**Table 3:** Sensitivity, specificity, and accuracy of PSA > 19.8 ng/mL, ISUP > 3, and SUVmax > 7.4 for the detection of metastasis on [^99m^Tc]Tc-HYNIC-ALUG SPECT/CT.

	Se	Sp	Ac
PSA>19.8 ng/ml	71.6%	76.9%	74.6%
ISUP>3	64.8%	66.9%	66.0%
SUVmax > 7.4	71.6%	82.6%	78.0%
PSA>19.8 ng/ml+SUVmax > 7.4	60.2%	84.3%	74.2%
ISUP>3+SUVmax > 7.4	58.0%	87.6%	75.1%

Se, sensitivity; Sp, specificity; Ac, Accuracy.

### Pelvic MRI and bone scan findings

Of the 227 patients, pelvic MRI and bone scans were available for 39 and 52 patients, respectively. As summarized in **Table 4**, positive findings were observed in 31 cases (79.5%) with pelvic MRI, while 23 cases (59.0%) showed positive results in [^99m^Tc]Tc-HYNIC-ALUG SPECT/CT imaging. Pelvic lymph node metastases were identified in 5 patients through MRI, with 3 of these cases confirmed by [^99m^Tc]Tc-HYNIC-ALUG SPECT/CT. Furthermore, for one patient with pelvic bone metastasis, both MRI and [^99m^Tc]Tc-HYNIC-ALUG SPECT/CT revealed metastases. Notably, [^99m^Tc]Tc-HYNIC-ALUG SPECT/CT identified additional bone metastases beyond the pelvic region. Among 17 high-risk patients, all had positive MRI results, and 16 were positive in [^99m^Tc]Tc-HYNIC-ALUG SPECT/CT imaging, with only one being negative. Among the remaining 22 low- and intermediate-risk patients, pelvic MRI detected 14 positives, while [^99m^Tc]Tc-HYNIC-ALUG SPECT/CT identified only 7 positives.

**Table 4:** Diagnostic results of pelvic MRI and [^99m^Tc]Tc-HYNIC-ALUG SPECT/CT on 39 PCa patients.

		MRI	PSMA SPECT/CT
Localization of positive lesions*	Prostate region	31 (79.5%)	23 (59.0%)
	Lymph node metastases	5 (12.8%)	3 (7.7%)
	Bone metastases	1 (2.6%)	1 (2.6%)
D’Amico risk group	Low	5 (12.8%)	2 (5.1%)
	Intermediate	9 (23.1%)	5 (12.8%)
	High	17 (43.6%)	16 (41.0%)

*PIRADS scores ≥3 were considered positive for the pelvic MRI.

Among the 52 patients who underwent bone scans, 4 cases exhibited diffuse bone metastases on both [^99m^Tc]Tc-HYNIC-ALUG SPECT/CT and bone scans. In the remaining 48 patients, 23 cases showed 41 positive bone lesions on [^99m^Tc]Tc-HYNIC-ALUG SPECT/CT, of which only 27 were visible on bone scans. Of the remaining 14 bone scan-negative lesions, 11 were bone marrow lesions, and 3 were osteolytic metastases. For the 25 patients with positive findings on bone scans, a comparative analysis was conducted with contemporaneous CT scans obtained from PSMA imaging. These positive findings were interpreted as degenerative changes, compromised bone fibrous structures, and evidence of previous fractures, without identification of typical or highly suspicious bone metastatic lesions.

## Discussion

The accurate initial staging of newly diagnosed PCa patients is essential for effective treatment planning and prognosis assessment. Nevertheless, conventional imaging modalities cannot accurately reflect the stage of tumors. The emergence of PSMA as a target for PCa diagnosis and treatment with radionuclides has sparked interest in the development of PSMA-targeted imaging agents. While PET tracers labeled with [^68^Ga]Ga, such as [^68^Ga]Ga-PSMA-11 and [^68^Ga]Ga-PSMA-617, have demonstrated excellent sensitivity for PCa staging, there is a notable lack of research concerning the utilization of [^99m^Tc]Tc-HYNIC-ALUG for the initial evaluation and staging of newly diagnosed PCa. To address this research gap, we conducted a study to investigate the role of [^99m^Tc]Tc-HYNIC-ALUG SPECT/CT in the primary staging of newly diagnosed PCa and its correlation with risk stratification parameters.

The results of this study demonstrated the potential of [^99m^Tc]Tc-HYNIC-ALUG in detecting primary tumors, achieving a positive detection rate of 92% among patients. These findings are consistent with previous research conducted by Werner et al. (18), Schmidkonz et al. (19), and Goffin et al. (20). In their respective studies, the use of a [^99m^Tc]Tc-labeled PSMA radiotracer for initial staging yielded primary tumor detection rates of 92%, 97%, and 94% in 11, 93, and 104 PCa patients, respectively. Regarding PSMA-targeted PET tracers, Basha et al. (21) reported a detection rate of 96% for [^68^Ga]Ga-PSMA-11 PET/CT in 173 patients with primary PCa. Uprimny et al. (22) and Sachpekidis et al. (23) demonstrated detection rates of 91% and 96% for primary tumor detection in 90 and 24 patients, respectively. In the largest [^68^Ga]Ga-PSMA-HBED-CC PET-CT study conducted by Yaxley et al. (24), overall detection rates of 95% were observed in 1253 patients. Moreover, pelvic MRI scans were performed on 39 patients in this study. The detection rates for MRI and [^99m^Tc]Tc-HYNIC-ALUG SPECT/CT in these 39 patients were 79.5% (31/39) and 59.0% (23/39), respectively. The lower detection rate of [^99m^Tc]Tc-HYNIC-ALUG SPECT/CT compared to MRI is primarily due to the fact that, among the 22 low- and intermediate-risk patients, the detection rate for [^99m^Tc]Tc-HYNIC-ALUG SPECT/CT was only 31.8% (7/22), whereas the pelvic MRI detection rate was 63.6% (14/22). In the initial diagnosis of low- and intermediate-risk PCa patients, the diagnostic performance of MRI is superior to [^99m^Tc]Tc-HYNIC-ALUG SPECT/CT, consistent with previous findings of [^68^Ga]Ga-PSMA-HBED-CC PET/CT (25).

Despite the encouraging detection rates achieved with [^99m^Tc]Tc- or [^68^Ga]Ga-labeled PSMA imaging, it is worth noting that some primary PCa tumors may not exhibit significant PSMA uptake. Schreiter et al. (26) attributed this to the lack of soft tissue contrast in CT scans during the initial staging of primary PCa, as well as the small size of the tumor, which may not show sufficient tracer uptake for effective detection. Additionally, Meyrick et al. (27) suggest that the absence of PSMA avidity may indicate a more aggressive form of the disease, such as high-grade/neuroendocrine PCa.

The early identification of metastatic disease in patients with PCa at the initial stage is pivotal for devising effective therapeutic strategies and averting unnecessary major surgical interventions. Our study results unequivocally demonstrate the efficacy of [^99m^Tc]Tc-HYNIC-ALUG SPECT/CT as a valuable and promising tool for detecting advanced disease. In our patient cohort, [^99m^Tc]Tc-HYNIC-ALUG SPECT/CT proficiently identified metastatic lesions in the lymph nodes, bones, and viscera in 30%, 26%, and 3% of patients, respectively. Notably, our study found that lymph nodes were the most prevalent sites of extraprostatic metastases. In comparison to previous studies by Schmidkonz et al. (20) (17%; 16/93) and Yaxley et al. (24) (9%; 107/1,253), our findings indicated a significantly higher percentage of both regional and nonregional nodal metastases. Additionally, we observed only one patient with nonregional nodal metastases without concurrent regional nodal metastases, which aligns with the traditional pattern of lymph node spread from the pelvis upward via regional lymphatic vessels (28).

Our study detected bone metastasis in 2% of patients with PSA levels <10 ng/mL and in 16% with PSA levels ranging from 10 to 20 ng/mL. A study by Yaxley et al. (24) reported a similar incidence of skeletal metastasis (15%) in the PSA level 10–20 ng/mL group using [^68^Ga]Ga-PSMA-HBED-CC PET/CT. Similarly, Klingenberg et al. (29) identified a comparable likelihood of detecting skeletal metastasis on [^68^Ga]Ga-PSMA-HBED-CC PET/CT, with a PSA level of <10 ng/mL at 8% and a PSA level of 10-20 ng/mL at 11%. In clinical practice, the [^99m^Tc]Tc-methylene diphosphonate bone scan has been conventionally employed for bone metastasis detection. Among the 52 patients who underwent bone scans, our study results indicate that [^99m^Tc]Tc-HYNIC-ALUG SPECT/CT is superior to bone scintigraphy in detecting bone metastases, as it can identify both bone marrow involvement and osteolytic metastases, consistent with previous findings from [^68^Ga]Ga-PSMA-11 PET/CT (30). Otherwise, our study reported an 18% detection rate of bone metastasis in PCa patients with a PSA level of <20 ng/mL, which is significantly higher than the 4.7% detection rate observed in a previous study of 857 consecutive PCa patients (31). Additionally, Falchook et al. (32) reported a markedly lower detection rate of <1% (4/11) in low- and intermediate-risk PCa patients. These results further substantiate the superior efficacy of [^99m^Tc]Tc-HYNIC-ALUG SPECT/CT in detecting skeletal metastases.

Furthermore, we demonstrate a correlation between advanced disease and rising PSA levels as well as ISUP grade. Additionally, we discerned a higher proportion of patients in the high-risk group than in the intermediate-risk group, with nodal (both regional and nonregional) and bone metastases. These findings align consistently with previous studies using PSMA PET/CT (8, 24, 33), which lends further validation to the role of [^99m^Tc]Tc-HYNIC-ALUG SPECT/CT in primary PCa staging.

In a subgroup of patients who underwent RP with concurrent PLND, we achieved a consistently high specificity of 93.6%, which is similar to the finding reported in a previous study (8). However, it is noteworthy that [^99m^Tc]Tc-HYNIC-ALUG SPECT/CT only detected 12 out of 51 histologically confirmed lymph node metastases in our patient cohort, resulting in a sensitivity of 23.5%. It is important to note that this subgroup had a significant selection bias, as patients scheduled for surgery are typically considered free of metastatic disease based on [^99m^Tc]Tc-HYNIC-ALUG SPECT/CT scans. Furthermore, we observed that the pathological sizes of metastatic lymph nodes detected by [^99m^Tc]Tc-HYNIC-ALUG SPECT/CT imaging were significantly larger than those in patients where no detection occurred. Considering the lower spatial resolution of SPECT/CT compared to PET/CT, the sensitivity observed in our study was lower than reported in other studies using [68Ga]Ga- or [18F]F-labeled PSMA PET/CT (33% to 42%) (8, 24, 34, 35). It should be noted, however, that a considerable proportion of patients still had undetected metastatic lymph nodes in [^99m^Tc]Tc-HYNIC-ALUG SPECT/CT imaging. Therefore, for patients undergoing RP, especially those at high risk, it is essential to perform PLND even when [^99m^Tc]Tc-HYNIC-ALUG SPECT/CT imaging does not detect metastatic lymph nodes.

Our study also examined the SUVmax of primary prostate tumors, which showed positive correlations with PSA levels and ISUP grade. Furthermore, we confirmed that the high-risk group had a higher SUVmax in their primary tumors than the low- and intermediate-risk groups. Similar correlations between higher SUVmax and increasing PSA levels and ISUP grade have been observed in studies using [^68^Ga]Ga-PSMA-11 (22, 23) and [^18^F]PSMA-1007 (36, 37). Additionally, we found that patients with nonadvanced disease had a significantly lower primary tumor SUVmax than those with advanced disease. This finding aligns with a study by Sathekge et al. (23), which reported that the SUVmax of the primary tumor was significantly higher in patients with extraprostatic metastases than in those without metastases.

The current EAU guidelines classify high-risk PCa patients based on PSA levels >20 ng/mL or ISUP grade >3. In our study, [^99m^Tc]Tc-HYNIC-ALUG SPECT/CT was used to assess PSA levels and SUVmax of primary prostate tumors in predicting metastatic disease. We determined an optimal cutoff value of PSA level >19.8 ng/ml for predicting metastatic disease, which yielded satisfactory sensitivity (71.6%) and specificity (76.9%). This cutoff for PSA levels aligns with the recommended parameters for PCa staging in the current EAU guidelines (38). For ISUP grade >3, the sensitivity for detecting metastatic disease using [^99m^Tc]Tc-HYNIC-ALUG SPECT/CT in our population was 64.8%, with a specificity of 66.9%. Additionally, we found that SUVmax >7.4 has good specificity (71.6%) and sensitivity (82.6%) in discriminating metastatic disease in our study. When combining SUVmax >7.4 with PSA >19.8 ng/ml, the sensitivity for detecting metastatic disease was 60.2%, with a specificity of 84.3%. Combining SUVmax >7.4 with ISUP >3 resulted in a sensitivity and specificity of 58.0% and 87.6%, respectively. SUVmax, when combined with PSA >19.8 ng/ml or ISUP grade >3, exhibits high specificity but reduced sensitivity, potentially leading to the missed diagnoses of some patients. Therefore, utilizing PSA >19.8 ng/mL or SUVmax >7.4 as selection criteria would be suitable for identifying the majority of patients with metastatic disease using [^99m^Tc]Tc-HYNIC-ALUG SPECT/CT for PCa staging.

Several limitations merit consideration in the current study. First, the retrospective nature of the study raises concerns about potential selection bias. Second, it is not practically possible to confirm every instance of extraprostatic avidity on [^99m^Tc]Tc-HYNIC-ALUG SPECT/CT through pathological examination. However, in the subgroup of patients who underwent subsequent RP with PLND, we performed a pathological examination of lymph nodes. Our findings revealed a sensitivity of 23.5%, a specificity of 93.6%, and an accuracy of 65.9% in detecting pelvic lymph node metastasis. Future research should focus on larger patient cohorts and prospective studies to further validate our findings.

## Conclusion

In conclusion, [^99m^Tc]Tc-HYNIC-ALUG SPECT/CT emerges as a promising imaging modality for the primary staging of 227 consecutive patients with newly diagnosed PCa. Its ability to detect advanced disease, assess lymph node and bone metastases, and predict disease aggressiveness based on PSA level and SUVmax offers valuable insights for clinical decision-making. This study highlights the potential of [^99m^Tc]Tc-HYNIC-ALUG SPECT/CT as an accessible and effective imaging tool, complementing the existing arsenal of diagnostic modalities for PCa, and holds promise for improving patient outcomes through better risk stratification and treatment planning.

## Data availability statement

The original contributions presented in the study are included in the article/supplementary material. Further inquiries can be directed to the corresponding authors.

## Ethics statement

The studies involving humans were approved by The Institutional Review Board of Henan Provincial People’s Hospital & Zhengzhou University People’s Hospital. The studies were conducted in accordance with the local legislation and institutional requirements. The ethics committee/institutional review board waived the requirement of written informed consent for participation from the participants or the participants’ legal guardians/next of kin because Informed consent was waived because of the retrospective nature of the study, and the analysis used anonymous clinical data.

## Author contributions

BL: Data curation, Formal analysis, Methodology, Software, Writing – original draft, Writing – review & editing. XD: Data curation, Formal analysis, Software, Writing – original draft. LD: Data curation, Formal analysis, Methodology, Software, Validation, Writing – original draft. JS: Data curation, Methodology, Validation, Writing – original draft. MT: Data curation, Investigation, Software, Visualization, Writing – review & editing. JZ: Data curation, Methodology, Visualization, Writing – review & editing. ZZ: Investigation, Software, Visualization, Writing – review & editing. XW: Data curation, Formal analysis, Methodology, Resources, Validation, Writing – original draft. YG: Conceptualization, Formal analysis, Funding acquisition, Methodology, Project administration, Validation, Writing – review & editing.
